# Commentary: Regenerative therapies for refractory thin endometrium in *in vitro* fertilization

**DOI:** 10.3389/fcell.2026.1763713

**Published:** 2026-03-19

**Authors:** Ezri Chow, Elean To, Tony K F Chow

**Affiliations:** 1 Reproductive Services Unit, The Royal Women's Hospital, Parkville, VIC, Australia; 2 Manningham General Practice Templestowe Lower, Melbourne, VIC, Australia; 3 Australia and New Zealand College of Anaesthetists, Melbourne, VIC, Australia

**Keywords:** clinical pregnancy, intrauterine platelet-rich plasma, meta-analysis, randomized controlled trial, thin endometrium

## Introduction

We read the narrative review by Chen and colleagues with great interest (Chen et al., 2025). In particular, we appreciate the detailed lists of case series, cohort studies, controlled clinical trials, and meta-analyses for each regenerative therapy. Although we agree that heterogeneity in study design, sample size, and outcome measures complicates direct comparisons, we believe that quantitative evaluations can offer further insights.

## Intrauterine platelet-rich plasma (PRP) in the management of thin endometrium

Consider intrauterine autologous PRP infusion as an example; a meta-analysis can be conducted using the exact data from the five controlled clinical trials referenced by Chen et al. (2025) to assess its effectiveness in managing thin endometrium on two primary outcomes: endometrial thickness on the day of embryo transfer and clinical pregnancy (Eftekhar et al., 2018; Chang et al., 2019; Nazari et al., 2019; Kusumi et al., 2020; Yu et al., 2024). The five trials were included according to Chen et al.‘s selection criteria, and the data were extracted from the results sections of the original articles. The extracted data were cross-checked against those in Chen et al.‘s Table 1 to ensure the reproducibility of our meta-analysis.

**FIGURE 1:**
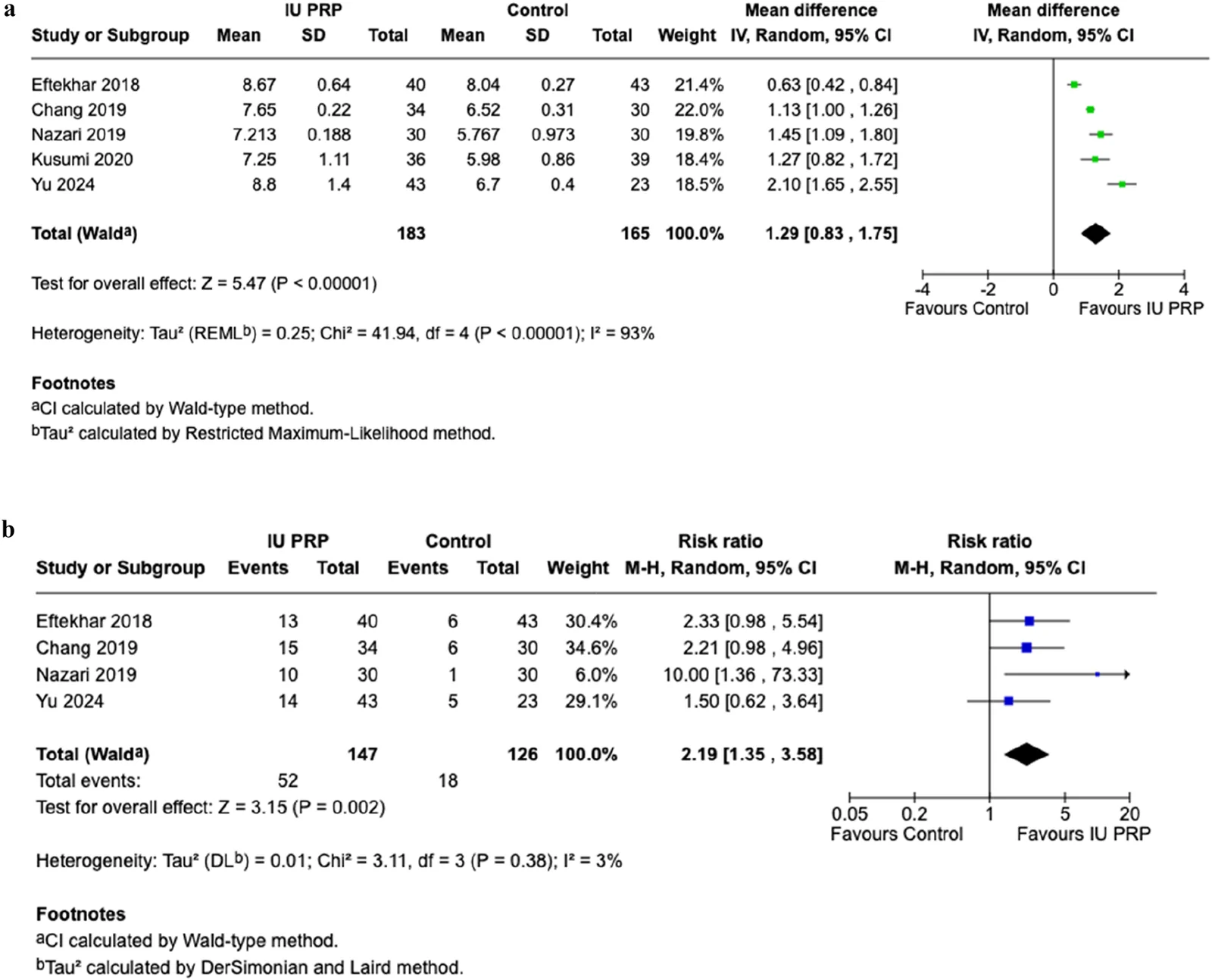
**(a)** Using data reported by Chen et al. (2025) to evaluate the effectiveness of IU PRP in managing endometrial thickness: Mean thickness on the day of embryo transfer. **(b)** Using data reported by Chen et al. (2025) to evaluate the effectiveness of IU PRP in managing endometrial thickness: Clinical pregnancy.

Endometrial thickness on the day of embryo transfer was reported in all five controlled clinical trials, including the mean thickness (mm), the standard deviation (SD), and the total number of patients. Notably, Kusumi et al. (2020) reported both unblinded and blinded values, and the unblinded measurement was included in our meta-analysis because the other four controlled clinical trials also reported unblinded measurements. Among 183 patients undergoing intrauterine PRP and 165 controls, the mean difference was 1.29 mm (95% CI: 0.83–1.75 mm), with an overall p-value of <0.00001. (Figure 1a). It is noteworthy that all five controlled clinical trials had small sample sizes, which may have contributed to significant statistical heterogeneity (I^2^ = 93%); however, they consistently favoured intrauterine PRP (Figure 1a).

Clinical pregnancy was reported in four controlled clinical trials (Figure 1b). Since clinical pregnancy is a dichotomous variable, the small sample sizes in the individual trials pose a clear issue: three of the four trials have 95% CI that includes 1, and therefore arguably are not statistically significant. This is where the advantage of a meta-analysis becomes evident. By combining the four controlled clinical trials, we demonstrate that 52 of the 147 patients (35.4%) who received intrauterine PRP achieved clinical pregnancy, compared with 18 of 126 controls (14.3%) who had a standard stimulation cycle. This resulted in a risk ratio of 2.19 (1.35-3.59), p = 0.002, which is statistically homogeneous (p = 0.38 and I^2^ = 3%) (Figure 1b).

## Discussion

This meta-analysis supports Chen et al.‘s narrative conclusions, but given the small sample size of the five trials, it remains inconclusive whether intrauterine PRP is effective in managing refractory thin endometrium. However, it highlights several key points. First, an average increase of 1.29 mm is both statistically significant and clinically meaningful, possibly influencing the decision to proceed with embryo transfer. Second, the favorable increased rate of clinical pregnancy with intrauterine PRP was associated with relatively wide 95% CI, supporting the need for further research, especially through larger randomized controlled trials. In conjunction with the detailed narrative summary by Chen and colleagues (Chen et al., 2025), we believe these findings bolster the case for human ethics approval and research funding to undertake such trials.
