# Correction: Commentary: Regenerative therapies for refractory thin endometrium in *in vitro* fertilization

**DOI:** 10.3389/fcell.2026.1912719

**Published:** 2026-08-10

**Authors:** Ezri Chow, Elean To, Tony K F Chow

**Affiliations:** 1 Reproductive Services Unit, The Royal Women’s Hospital, Parkville, VIC, Australia; 2 Manningham General Practice Templestowe Lower, Melbourne, VIC, Australia; 3 Australia and New Zealand College of Anaesthetists, Melbourne, VIC, Australia

**Keywords:** clinical pregnancy, intrauterine platelet-rich plasma, meta-anaiysis, randomized controlled trial, thin endometrium

Affiliation 1 “Reproductive Services Unit, The Royal Women’s Hospital, Parkville, VIC, Australia” was erroneously given as “Public Fertility Care, The Royal Women’s Hospital, Parkvielle, VIC, Australia”.

Author Tony Chow was erroneously assigned to affiliation “Public Fertility Care, The Royal Women’s Hospital, Parkville, VIC, Australia”. This affiliation has now been removed for author Tony Chow.

Affiliation “Australia and New Zealand College of Anaesthetists, Melbourne, VIC, Australia” was omitted for author Tony Chow. This affiliation has now been added for author Tony Chow.

An incorrect email address was provided for the corresponding author. The correct correspondence email address is: tonykfchow@gmail.com.

The original version of this article has been updated.

